# Protein and Polysaccharide-Based Electroactive and Conductive Materials for Biomedical Applications

**DOI:** 10.3390/molecules26154499

**Published:** 2021-07-26

**Authors:** Xiao Hu, Samuel Ricci, Sebastian Naranjo, Zachary Hill, Peter Gawason

**Affiliations:** 1 Department of Physics and Astronomy, Rowan University, Glassboro, NJ 08028, USA; riccis1@students.rowan.edu (S.R.); hillza84@students.rowan.edu (Z.H.); 2 Department of Biomedical Engineering, Rowan University, Glassboro, NJ 08028, USA; naranjos1@students.rowan.edu (S.N.); gawasonp1@students.rowan.edu (P.G.); 3 Department of Molecular and Cellular Biosciences, Rowan University, Glassboro, NJ 08028, USA

**Keywords:** protein, polysaccharide, electroactive material, tissue regeneration, drug delivery and nanomedicine, biosensor

## Abstract

Electrically responsive biomaterials are an important and emerging technology in the fields of biomedical and material sciences. A great deal of research explores the integral role of electrical conduction in normal and diseased cell biology, and material scientists are focusing an even greater amount of attention on natural and hybrid materials as sources of biomaterials which can mimic the properties of cells. This review establishes a summary of those efforts for the latter group, detailing the current materials, theories, methods, and applications of electrically conductive biomaterials fabricated from protein polymers and polysaccharides. These materials can be used to improve human life through novel drug delivery, tissue regeneration, and biosensing technologies. The immediate goal of this review is to establish fabrication methods for protein and polysaccharide-based materials that are biocompatible and feature modular electrical properties. Ideally, these materials will be inexpensive to make with salable production strategies, in addition to being both renewable and biocompatible.

## 1. Introduction

Biopolymers are renewable materials created by or derived from living things [1]. This classification is a broad category including a variety of different material classes, with proteins [2,3], polysaccharides, and nucleic acids all being considered types of biopolymers [4]. Being particularly versatile, energy storage [5], thin film transistors [1], electroluminescence [1], tissue engineering [6,7,8,9,10,11], biosensing [12], wound-healing [8,13,14,15], and drug delivery [3,13,16,17,18] are all areas wherein biopolymers are being utilized. While nucleic acids do fall under the category of biopolymers, this review will focus on proteins and polysaccharides which have been widely studied recently as biomaterials. Biopolymers offer novel and useful alternatives to traditional metal and synthetic polymer-based materials. These traditional materials, when used in biomedical devices, suffer from mechanical strain and resistance from the surrounding tissue in vivo [19]. Biomaterials, sensitive to electrical changes, offer the potential to overcome these limitations considering the materials will adapt to their surrounding as necessary.

In particular, proteins and polysaccharides have gained considerable traction as potential sources of biomaterials for electrical applications in medicine and beyond. Biopolymers of the protein family show traits such as, low cost [13], high availability [8,20,21], and biodegradability [3,10]. Established protein sources include silk [22,23,24,25], keratin [10,11,20,21,26], collagen [27], elastin [28], zein [29], soy [30], reflectin [31], and hybrid-protein mixtures [32]. Proteins are natural and therefore safe for biomedical applications, making them a strong candidate. Although, a well-documented limitation of protein-based biomaterials is temperature sensitivity. On the other hand, polysaccharide biopolymers are structurally flexible [33], hydrophilic [33], biodegradable [34], and abundant worldwide [33,34]. Many polysaccharides, including cellulose [35], starch [36], chitin, and chitosan [7,37] are remarkably stable but are less biocompatible than proteins. Antimicrobial properties have also been noted amongst some biopolymers, including chitosan [14]. In essence, synthetic materials show reduced biocompatibility [2] with more environmentally harmful properties compared to biopolymers, with additional dependences on petroleum availability [34]. The emergence of combination protein-polysaccharide biomaterials provides a new standard of biomaterials capable of combining the biocompatibility of proteins and structural stability of polysaccharides [38].

One particularly promising aspect of biopolymers is their electrical properties. In general, electrical current requires the flow of charge carriers through a material—electrons, for example [39,40,41]. The energy levels of electrons in the material determine how well an electron current can be carried through the material [42]. A sizable energy gap between the valence and conductance bands makes a current difficult to sustain, making the material an insulator [42]. A small energy gap makes a current sustainable when the material is subject to an external electric field, making the material a semiconductor [42]. An overlap in energy levels makes current intrinsically easy to maintain, making the material a conductor [42].

Natural materials are created and safely utilized for a variety of tasks necessary for survival. Blood circulation [43], tissue regeneration, vision, and metabolism are a few examples wherein an organism depends upon the electrically conductive biopolymers that it produces [44]. These biopolymers can be artificially put to other uses as well, including printable electronic circuitry [25,41] and light-emitting transistors [39]. Further advances in biopolymer science can drastically increase the usefulness of such biopolymer-based circuitry, making biomedical devices far safer and more versatile.

The purpose of this review is to extensively analyze the characteristics, properties, and fabrication techniques of different protein and polysaccharide-based materials and elucidate their role in electrical applications for several biomedical and engineering purposes. First, an overview of various proteins and polysaccharides is provided that introduces the background of each biopolymer and explains its characteristics and molecular properties. Protein and polysaccharide composite materials and their advantages over singular biopolymer materials will also be examined. Subsequent sections will elaborate on electrical material theory and factors of electrical property and describe types of fabrication techniques/methods for these materials. Lastly, a section will discuss multiple electrical applications for protein and polysaccharide-based biomaterials, such as nanomedicine, drug delivery, tissue regeneration, and biosensors.

## 2. Types of Protein and Polysaccharide Materials

There are numerous proteins and polysaccharides analyzed in modern-day experimentation and used for a wide variety of purposes including mechanical, electrical, and chemical applications. This review will focus particularly on protein and polysaccharide biopolymers commonly used in electrical applications. In terms of composition, proteins are macromolecules made up of long-chain amino acids while polysaccharides are carbohydrates comprised of multiple long-chain bonded sugar molecules. The proteins examined in this section include silk, keratin, soy, collagen, elastin, zein, and reflectin while the polysaccharides examined were cellulose, chitin, chitosan, alginate, and starch. Background information on each biopolymer is provided as well as an in-depth overview of the intrinsic/natural properties for each material.

### 2.1. Typical Protein Biopolymers

#### 2.1.1. Silk

Silk is a natural fibrous protein polymer secreted from animal organisms. Nearly four hundred species of insects are known to generate silk, which is used to anchor, entangle prey, or form protective sheaths. Silk proteins are usually produced in specialized glands after biosynthesis in epithelial cells, and then secreted into the lumen of these glands, where the proteins are stored before spinning into silk fibers for use. Silk fiber is a semi-crystalline polymer, dominated by ordered β-sheet secondary structures. One such type of silk protein that has been explored is spider silk. Spider silk fiber has been used for its unique properties, such as its versatility and remarkable strength [13].

Silk proteins have been exploited recently in a wide range of biomaterials such as films, hydrogels, microspheres, nanoparticles, nanofibers, or different micro-/nano-patterned devices for various biomedical applications. The unique mechanical and chemical properties of these fibers have provided important clinical treatment options for many future scopes. Silk also has good biocompatibility and excellent thermal properties when used in a biomaterial but has shown inconsistent results when included for electrical uses due to its questionable conductivity [45].

#### 2.1.2. Keratin

Keratin is a group of proteins that makes up the skin, hair, and nails. Keratin can also be found in the internal organs and glands. Keratin is a protective protein with coiled-coil/helix structures, less prone to scratching or tearing than other types of cells. Keratin materials can be derived from the animal wool, feathers, horns, and human hair. Since keratin is the structural building block of hair, it is believed that keratin products, supplements, and treatments can help strengthen the hair fibers and make them look healthier [46].

#### 2.1.3. Soy

Soy protein is a type of globular protein with two main subunits, 35% of conglycinin (7S) and 52% of glycinin (11S). Soy protein is low-cost, of non-animal origin, and has relatively long storage time and thermal stability, which has advantages over other natural proteins utilized for biomedical applications. The intrinsic physicochemical properties of soy can affect the behavior of protein in food systems during processing, manufacturing and storage, such as solubility, ligand-binding, sorption, gelation and film formation. These characteristics reflect the conformation and composition of the soy proteins, as well as their interactions with other food components, which are also affected by processing treatments and the environment [13,30,47].

#### 2.1.4. Collagen

Collagen is the main structural protein with a triple-helix structure found in the extracellular matrix of various connective tissues such as cartilage, bones, tendons, ligaments, and skin. It is the most abundant protein in mammals, accounting for 25~35% of the protein content in the whole body. A single collagen molecule is composed of over 1000 amino acids, which are bounded together to form three elongated α-helix fibril chains, known as a collagen helix [48].

#### 2.1.5. Elastin

Elastin is a main extracellular matrix (ECM) protein with dominated β-spiral structures that provides elasticity and resilience to tissues and organs. Elastin can be 1000 times more flexible than collagens; therefore, the main function of elastin proteins in extensible tissues (such as lungs, aorta, and skin) is the elasticity of tissues. Due to its key role in the normal development and function of vital organs, either impaired elastin synthesis or proteolytic degradation of the insoluble fibers leads to major clinical pathologies [49].

#### 2.1.6. Zein

Zein is one of the best-understood plant proteins found in the endosperm tissue of corn. Pure zein is clear, water-insoluble, hard, odorless, and tasteless and has a variety of industrial and food uses. The tertiary structure of corn zein protein involves nine topologically antiparallel and adjacent helix structures with hydrophilic ends. Due to its high proportion of nonpolar amino acid residues, zein has exhibited a high degree of hydrophobicity and insolubility characteristics. Biodegradability and biocompatibility are the main advantages of this protein material. These properties make it a good candidate for use in different biomedical fields [50].

### 2.2. Typical Polysaccharide Biopolymers

#### 2.2.1. Cellulose

Cellulose is a crystalline polysaccharide appearing as an odorless, white powdery fiber. It is the most abundant carbohydrate present in nature and is found as a linear polymer composed of long chain glucose units. Cellulose is also the biopolymer composing the cell wall of vegetable tissues. Microcrystalline cellulose can be extracted by treating cotton fibers with organic solvents to de-wax it and then removing pectic acids through a solution of sodium hydroxide [35].

#### 2.2.2. Chitin

Chitin is a translucent, pliable, resilient, and quite tough polysaccharide with the N-acetylglucosamine units. As one of the most important biopolymers in nature, chitin is mainly produced by fungi, arthropods, and nematodes. Chitin functions as scaffold material in insects, supporting the cuticles of the epidermis and trachea as well as the peritrophic matrices lining the gut epithelium. It is often modified as a component of composite materials in most arthropods, such as in sclerotin, which forms most of the exoskeleton of insects (as a tanned proteinaceous matrix) [44].

#### 2.2.3. Chitosan

Chitosan is a sugar-like polysaccharide made up of both 1–4 linked 2-acetamido-2-deoxy-β-D-glucopyranose as well as 2-amino-2-deoxy-β-D-glucopyranose. Chitosan can be obtained from the hard-outer skeleton of shellfish, such as shrimp, crab, and lobster. Chitosan is non-toxic and has good biodegradability and antifungal effect, which accelerate wound healing and stimulate the immune system. It is commonly used in a variety of medicines for high blood pressure, high cholesterol, obesity, wound healing, and other conditions. As the deacetylated form of chitin, chitosan has several biological properties that make it a highly attractive material for use in biomedical applications, especially when dealing with skin or bone [7,51].

#### 2.2.4. Alginate

Alginate is a naturally occurring anionic polymer derived from brown seaweed, containing blocks of (1,4)-linked β-D-mannuronate (M) and α-L-guluronate (G) residues. Due to its biocompatibility, relatively low cost, and mild gelation by addition of divalent cations, alginate has been extensively investigated for many biomedical applications [52]. The main industrial applications of alginate are related to its stabilizing, viscosifying, and gelling properties and its water retention capacity. Alginate is also largely used as a viscosifier in textile printing industry because of its shear-thinning properties.

#### 2.2.5. Starch

Starch is the second largest biomass produced on earth, created as energy storage molecules for plant life [53]. Pure starch is mainly composed of two types of molecules: the linear and helical amylose and the branched amylopectin. Starch powder is white, tasteless, odorless, and insoluble in cold water or alcohol. The functional properties of starch granules include swelling power, starch solubility, gelatinization, retrogradation, syneresis, and rheological behavior, which are generally determined by the multiple characteristics of starch structure. Due to its intrinsic thermal properties, the functional properties of starch are determined by the measured change of heat during its gelatinization process.

## 3. Theory

**Figure 1:**
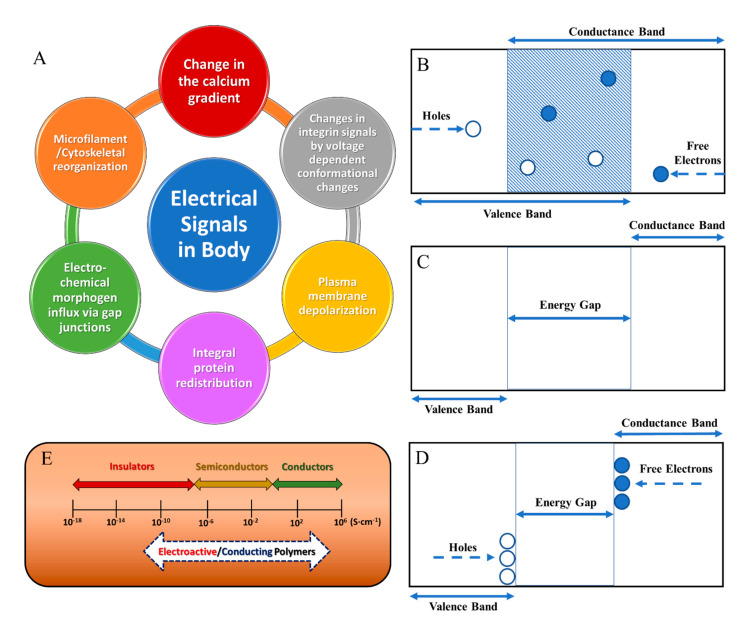
(**A**) Different electrical signals utilized in the body. Energy gap representations for (**B**) conductors, (**C**) insulators, (**D**) semiconductors, and (**E**) conductivity ranges for these material classifications compared to typical conducting/electroactive polymers.

The body utilizes electrical properties for a variety of tasks (Figure 1A). Neurons, in the presence of electrical stimulation, open the voltage gated channel responsible for the influx of calcium—and thus undergo neural regeneration and cell birth [54,55,56]. Cardiomyocytes have also been shown to be electrically active cells, through which electrical signaling coupling through gap junctions are responsible for the heart’s contractions [57,58]. The heart tissue has also been shown to work in conjunction with bone tissue in producing these contractions [43,59]. Even bone cells will experience electrical stimulation due to the stress exerted onto it by muscle contractions [60]. Electrical field stimulation, furthermore, modulates numerous stem cell functions, including mechano-sensing and the activation of signaling pathways associated with stem cell differentiation [61]. The body’s electrical properties, therefore, are a vital part of sustaining life.

Materials are classified into three subtypes of materials based on the configuration of the conductance and valence electron bands. The conductance band contains excited electrons, and the valence band contains excitable electrons [42]. The difference in energy levels between the two bands is the distinguishing factor between the conducting, insulating, and semiconducting materials [42]. Conducting materials are materials in which their conducting and valence electrons bands overlap and allow for the flow of electrons in the presence of an external electrical fields (Figure 1B). Juxtaposed to conducting materials, insulators resist the flow of electrons because of the relatively large distance between bands (Figure 1C). Semiconducting materials contain a discrete distance between conductance and valence electron bands, thus allowing for conductivity as a function of the number of free charge carriers with respect to the external electrical field present (Figure 1D). The conductivity ranges for these material classifications comparing to typical conducting or electroactive polymers are shown in Figure 1E.

In general, the conductance (*G*) of these materials is defined as the reciprocal of the resistance (*R*) (Equation (1)), which is the materials’ ability to resist the flow of charge (Equation (2)).

(1) $$G=\frac{I}{R}$$

(2) $$R=\frac{V}{I}$$

where *V* is the applied voltage, *I* is the current, and the material resistance *R* is derived from Ohm’s law (Equation (2)). Electroactive polymers are further divided into two subclasses: polymer electrolytes and conducting polymers. Polymer electrolytes conduct electricity via ion deposition and interfacial ions through the solidification of ions, therefore facilitating the induction of ions flow across matrix electrochemical reactions and material conductance. Conducting polymers are intrinsically conductive because double conjugated π bonds are responsible for their electrical conductivity phenomena [62]. Ion conducting materials assume that the polymeric matrix conductance is independent or negligible from the bulk properties as a means of the system simplification in order to assume that the bulk conductance is exclusively dependent on time and electrochemical reactions in the matrix as a function of the electrolytic species [42] (Equation (3)).

(3) $$\sigma \left(T\right)=\sum n_{i}q_{i}\mu_{i}$$

where the materials intrinsic conductance (σ) at a specific temperature (*T*) is computed as the summation of product between number of charged specific per mole (*n*), the overall charge of each ion (*q*), and the ion mobility of each species (*µ*). The electrical fields experienced by theses ion-conducting species are associated to the changes in electrical gradients associated red-ox reactions occurring in the presence of an external voltage.

Special materials like collagen, cellulose, and silk fibers exhibit intrinsic piezoelectric behavior, where electric polarization occurs perpendicular to the mechanical stress experienced by the material [63]. The piezoelectric effect experience by cellulose nanocrystal thin films is attributed to the unevenly distributed carbon atoms and fluctuations in polarization density of charged atomic groups under electric fields associated with the anisotropic triclinic and monoclinic unit crystal structure. The vertical displacement (*D*) due to the application of an external voltage can be calculated by (Equation (4)) [63].

(4) $$D_{i,j,k}=d_{i,j,k}\sigma_{i,j,k}$$

where *d* is the piezoelectric constant based on the material properties, and the σ is the tensile stress. The displacement (*D*) experienced by the cellulose can be further used to calculate the induced charge (*q*) of the material (Equation (5)),

(5) $$q=\iint D_{i,j,k}dA_{i,j,k}$$

where the d*A* is an infinitesimal area of the material. The resultant voltage (*V*) generated by the material is then calculated by the induced charge (*q*) and material capacitance (*C*) (Equation (6)),

(6) $$V=\frac{q}{C}=\frac{q}{\frac{\varepsilon_{r}\varepsilon_{0}lw}{t}}$$

where the relative permittivity of the biopolymer ($\varepsilon_{r}$), the vacuum permittivity ($\varepsilon_{0}$ = 8.85 × 10^−12^ F/m), the film length (*l*), film width (*w*), and film thickness (*t*) are used to calculate the capacitance [63].

In intrinsically conductive polymers, the resistivity (*R*, related to shape, thickness, etc.) can be modified to produce the desired field parameters (Equation (7)),

(7) $$R=\frac{\rho L}{A}$$

where ρ is the material resistivity, *L* is the length, and *A* is the cross-sectional area.

However, in the presence of an alternating current (AC), the assumption of ideal polymeric behavior can no longer be applied to the system. In addition, materials’ impedance (*Z*) or effective resistivity is calculated by (Equation (8)), typically through electrochemical impedance spectroscopy (EIS) [64].

(8) $$Z=\frac{V}{I}=Z_{0}e^{j\phi }=Z_{0}\left(cos\phi +jsin\phi \right)$$

The real portion of the function corresponds to the resistance of the material, and the imaginary response is related to the material capacitance [42]. However, unless the material experiences capacitive coupling, the imaginary portion of the equation is neglected, and the impedance is calculated by the material’s resistivity. Even though the capacitance of a material is usually neglected in electroactive materials, the induction of an electrical field via capacitive coupling has been shown to have certain advantages over direct current stimulation: increased specificity and noninvasive nature.

Finally, the resultant electric field between the capacitor plates (*E_in_*) can be calculated as a function of charge density (σ) (Equations (9) and (10)).

(9) $$\sigma =\frac{Q}{A}=\frac{\varepsilon_{0}\varepsilon_{r}}{d}V_{g}$$

(10) $$E_{in}=\frac{V_{g}}{d}=\frac{\sigma }{\varepsilon_{0}{\varepsilon_{r}}^{\prime }}$$

where *V_g_* is the applied external voltage, $\varepsilon_{0}$ is the absolute permittivity, $\varepsilon_{r}$ is the relative permittivity of the dielectric medium between the plates, *Q* is the charge on the plates, *d* is the distance between the two plates, and *A* is the area of the plates. In addition, the electrical field penetrating past the capacitor plates can be obtained from (Equations (11) and (12)),

(11) $$E_{out}=\frac{V_{g}}{2\varepsilon }=\frac{\sigma^{\prime }}{2\varepsilon_{0}{\varepsilon_{r}}^{\prime }}$$

(12) $$\sigma^{\prime }=\frac{Q^{\prime }}{A}=\frac{\varepsilon_{0}{\varepsilon_{r}}^{\prime }V_{g}}{8\pi d}$$

where $E_{out}$ is the electrical field penetrating past the capacitor plate, *V_g_* is the applied external voltage, $\sigma^{\prime }$ is the induced carrier density on the material facilitating the penetration of the electrical field through the capacitive plates, and ${\varepsilon_{r}}^{\prime }$ is the relative permittivity between the plate material and the surrounding medium.

## 4. Fabrication Methods

Some biopolymers can be used in an almost raw state, as in the case of the experiments run by Tulachan et al. [5], where they managed to create crude proof-of-concept circuits to operate small red and blue LED lights using silk cocoons exposed to humidity [5]. Most applications, however, will require more processed forms of the materials. While more specific details will be given for individual processing methods below, it should be noted that many biopolymers require some amount of preparation prior to actual production. Some proteins such as silks need to be purified, boiled in saline, dried, and subsequently rinsed with pure water prior to use—a process called degumming—so that unwanted contaminants are removed from production materials [65]. Many fabrication methods will also require that the biopolymer be dissolved into a solution beforehand.

### 4.1. Spinning

**Figure 2:**
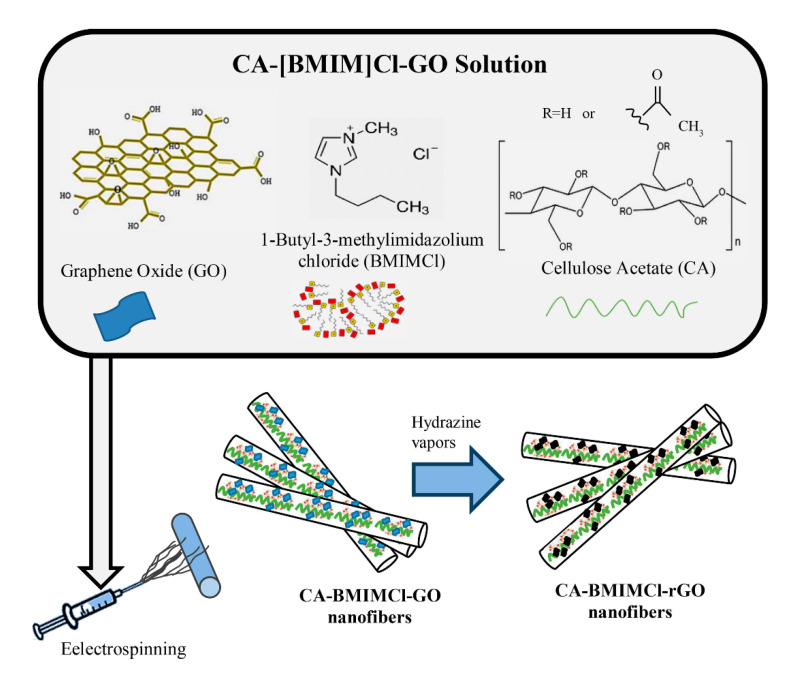
An electrospinning system combined cellulose acetate (CA), graphene oxide (GO) nanoparticles and 1-butyl-3-methylimidazolium chloride ([BMIM]Cl) to create conductive CA-[BMIM]Cl-GO nanofibers. The nanofibers were then converted to reduced GO nanofibers using hydrazine vapor to enhance the conductivity of the composite nanofibers [67]. Reproduced with permission from Elsevier, 2018.

Electrospinning is an innovative and low-cost technology that uses electrical forces, at a high voltage, to produce continuous polymer nanofibers [9,15,66]. It can be applied to either synthetic or natural polymers [67]. The procedure for electrospinning is composed of three main parts: the spinneret (such as a metal tip), a collection plate, and a high voltage power supply [66]. A polymer solution is sent through the spinneret and forms a drop at the very tip, held by surface tension [67]. Next, a high potential difference is established between the grounded collection plate and the spinneret [21]. Then once the surface tension is overpowered by the electric field, a charged polymer solution jet is emitted from the tip. As the jet travels, the solvent evaporates leaving the polymer fabric behind [8,21]. Using multiple spinnerets at the same time can increase production rates, provided that adequate spacing between the needles is available, lest the paths of the ejected solvent be bent by the electric forces between the jets [68]. Figure 2 demonstrated an electrospinning system using cellulose acetate (CA), graphene oxide (GO) nanoparticles and 1-butyl-3-methylimidazolium chloride ([BMIM]Cl) as the ionic liquid (IL) to generate conductive CA-[BMIM]Cl-GO hybrid nanofibers.

Several variants of the basic spinning method exist in addition to the basic solution electrospinning previously described, and a few related to biopolymer materials fabrication are highlighted here. Substituting the dissolution step with liquification yields a variant known as melt electrospinning, which does not suffer from impurities from potentially retained solvents but requires a biopolymer which is not denatured via melting and a setup which has the heating source near enough to the spinneret to avoid re-solidification within the spinneret [69]. A fiber can be spun with one biopolymer being completely enveloped by another through coaxial electrospinning, which differs from the base system by having two solvent chambers and two concentric nozzles [70]. Omitting the potential difference altogether, thus sparing those proteins or polysaccharides which are too fragile to withstand them without denaturing, can be achieved with dry-jet wet spinning—where a pump is used to eject the solution downwards through the air into a bath, from which the fibers are drawn and dried after the solvent diffuses out of the biopolymer [71]. While in principle it is possible to pump the solution directly into the bath rather than through the air, doing so will reduce the fiber’s molecular alignment—without significantly simplifying the production system [71].

These spinning methods can be implemented to improve many different biomedical applications, such as drug delivery, tissue engineering scaffolds, assistance in wound healing, and creating biosensors [10,11,12,69,70,71]. There are also other miscellaneous applications like cosmetics, producing protective clothing, and generating energy [15,18,24,25,72].

### 4.2. Crosslinking

**Figure 3:**
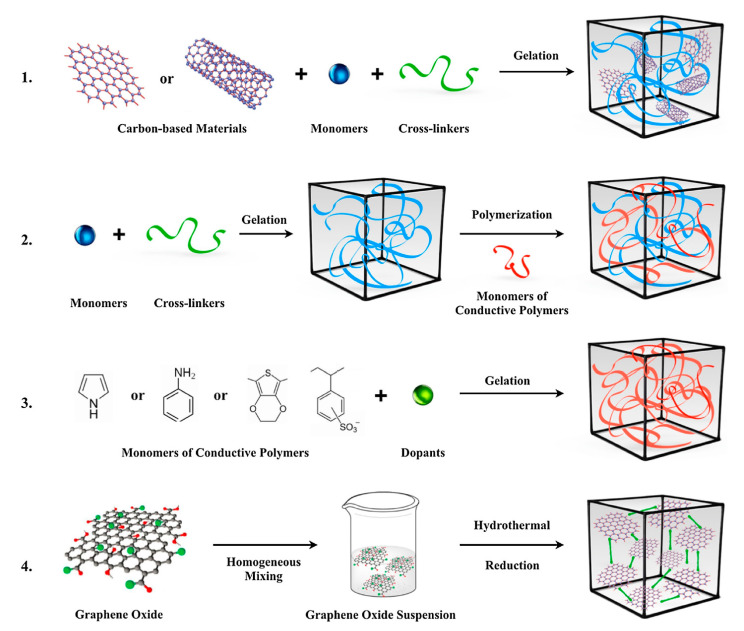
Typical crosslinking methods used to obtain conductive polymer network [74]: (**1**) formation of hydrogel network from polymer monomers, crosslinkers, and conductive fillers; (**2**) polymerization of conductive polymer monomers into a preformed hydrogel matrix; (**3**) crosslinking conductive polymers using dopant molecules; (**4**) self-assembly of conductive hydrogels using supramolecular interactions. Adapted with permission from Elsevier, 2019.

Crosslinking is an effective approach to introduce conductive fillers, polymer chains, or side chain groups into protein or polysaccharide molecules, which can provide biopolymer materials with new or enhanced electrical properties (Figure 3). Crosslinking can be either a physical or chemical process in which a branched or crosslinked biopolymer network is synthesized [73,74]. The yielded biopolymer exhibits its constitutive chemical composition while adopting alterations in its intrinsic physical properties (density, melt viscosity, crystallinity, etc.), as a function of the degree of branching/crosslinking [64]. Cross linkage can result from either physical or chemical interactions present between monomer chains [33].

Physical crosslinking is driven by electrostatic (-π) interactions, hydrogen bondings, chain entanglements, etc. and is therefore known as a potentially reversible process [64]. There are three major types of physical crosslinking: (i) point crosslinks, (ii) junction zones, and (iii) fringe micelles [64]. Point crosslinks are defined as the crosslinking of monomers at a single point [64]. Junction zone cross links form ordered secondary structures because the interactions between the two polymers are present throughout the entire length of the biopolymer segment [64]. The production of small crystallite domains is known as fringe micelles, where the alignment of small portions of the biopolymer chains result in microscopic crystalline regions [64].

Chemical crosslinking occurs when chemical bonds (intermolecular or intramolecular) are formed to interconnect monomers [33]. Intermolecular crosslinking is defined as the bonding of two functional groups on separate biopolymer molecules, resulting in the formation of a larger single polymer chain [75]. Intramolecular crosslinking occurs when two functional groups within the same biopolymer molecule form a connective loop, known as cyclization [64]. There are two types of cyclization that can occur: (i) primary cyclization, where a cycle is formed via the bonding of two functional groups of the same primary chain, and (ii) secondary cyclization, where a link between two already crosslinked primary chains is formed by the two functional groups attached [64]. This type of crosslinking results in a more compact biopolymer network attributed to the formation of crosslinks within the same primary chain [73]. The key parameters to control the intermolecular/intramolecular biopolymer crosslinking rates are the polymer concentration, crosslinker structure, and crosslinker content [64].

There are four different types of crosslinking polymerization: (i) step-growth, polymerization (ii) vulcanization, (iii) free radical copolymerization, and (iv) end-linking [64]. Step-growth polymerization occurs when bifunctional or multifunctional biopolymers dimerize, eventually forming long polymer chains [64]. Vulcanization is another way of chemically cross linking linear biopolymers through the attacking of active backbone functional groups with other agents (sulfur) [64]. This type of crosslinking is mainly used to improve the mechanical properties associated with the original biopolymer [76]. Free radical polymerization begins as a suitable material is broken down in solution to form free radicals [77]. These radicals react with nearby monomers, forming chains as more and more monomers are linked together, until diffusion effects bring two radicals together—linking the two chains and ending polymerization [73]. The product biopolymer chain is known to be heterogeneous structures due to their dependance on slow initiation rates, fast chain propagation, fast termination rates, and high molar mass dispersity of the primary chain [64]. The last type of crosslinking is known as end-linking, where two f-functional groups are added onto the ends of the linear biopolymer and then bonded together [64]. NorHA-dithiothreitol (DTT) crosslinking is an example of a radical mediated, vulcanization, and a step growth crosslinking process, where a photo initiator begins NorHA (hyaluronic acid functionalized with norbornene groups) polymer chain crosslinking through thiol bonds (S-H) [78].

Regardless of method chosen, the biopolymer networks (such as hydrogels) created are useful in both drug-delivery and tissue scaffolding applications with novel electrical properties in addition to their ability to encapsulate cells, their mechanical stability, and their hydrophilic nature [74].

### 4.3. Phase Separation

**Figure 4:**
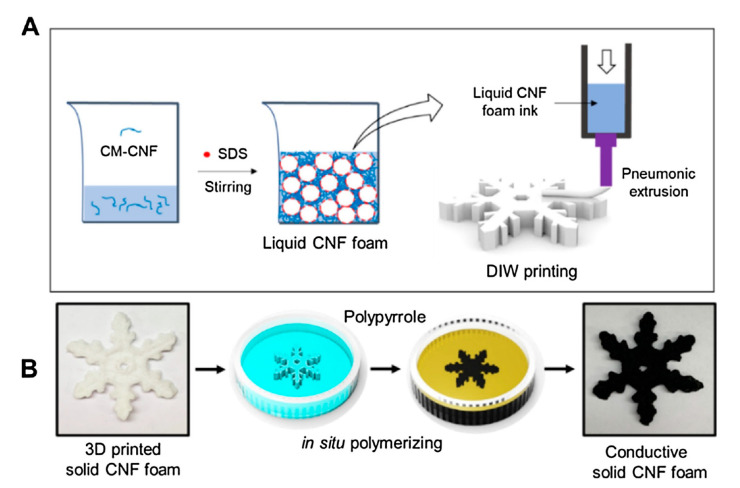
Procedure to prepare conductive solid cellulose nanofiber (CNF) foam-based 3-D materials [80]. (**A**) The liquid CNF foam ink is generated by stirring carboxymethylated (CM)-CNF suspension using sodium dodecyl sulfate (SDS) as an emulsifier to induce phase separation; (**B**) conductive foams are prepared through in situ polymerization of polypyrrole (PPy) on solid CNF foams. Adapted with permission from Elsevier, 2021.

To perform a phase separation, the desired biopolymer needs to be within a chemical solvent solution (Figure 4) [71,79,80]. Treatment of the solution will induce gel formation, after the gel is submerged in pure water to remove the solvent [68,71]. The gel can be freeze-dried after being removed from the water, creating a matrix of nanoscaffolds—the nature of which can be modified by varying biopolymer concentration and temperature in the initial solution [71,79,81]. This process generates a three-dimensional porous structure with only a few simple steps and can be used as tissue scaffolding—as the consistency of the matrix produced is very similar to the collagen extracellular matrix of the human body—but currently suffers from limited fiber control [67,81]. Despite this, the nanoscaffolds have good cell response and distribution characteristics, in addition to being easily grown in a mold to form the desired shape [67]. Furthermore, in electronic devices, it is crucial for the material to have the proper morphology to function. For example, phase separation of a binary blended polymer allows for the construction of continuous interconnected regions of each phase. This layout is important for several electronics including solar panels and LEDs [82].

### 4.4. Coatings

**Figure 5:**
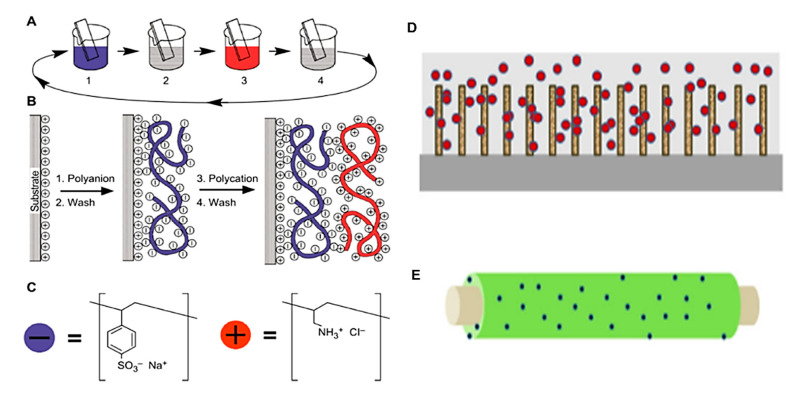
(**A**–**C**): Schematic of a layer-by-layer film coating procedure; steps 1 and 3 represent the adsorption of a polyanion and polycation, respectively, and steps 2 and 4 are washing steps [82]. (**D**,**E**): Examples of film casting and dip coating [4]: (**D**) cellulose cast from room temperature ionic liquids (spheres) onto multi-walled carbon nanotubes (cylinders) grown on silicon wafer; (**E**) dip-coating in lactose-modified chitosan and embedding Ag nanoparticles (spheres) on the thermoset core. (**A**–**C**) are reproduced with permission from Science, 1997. (**D**,**E**) are reproduced with permission from Elsevier, 2014.

Film casting is a relatively simple process used to create two-dimensional sheets of various dimensions (Figure 5). A thin film may be produced by pouring a solution of the desired biopolymer into a surface or mold of the desired shape and subsequently drying the solution in a fume hood, leaving the biopolymer behind [72,83,84]. Rendering the biopolymer water-insoluble afterwards via chemical treatment—such as with an organic solvent—will create a stable protein material for use within the body [84]. Adding glycerol to the solution when film casting with polysaccharides will improve the mechanical properties of the resulting polymer [4]. This process can be applied to a substance placed within the solution as well—creating a film about the desired object—a process known as dip coating [4]. In addition, the flexibility of thin film proteins makes them suitably for application in elastic areas of the body—such as on skin—making them more viable than rigid substates such as silicon in electrical systems in these areas [65,85]. Energy storage is another potential application, in the case of thin film polysaccharides [4]. Such films may also be utilized as scaffolding and cell-adhesion sies in tissue regeneration [84].

A three-dimensional structure can also be formed in a similar manner in a process referred to as layer-by-layer assembly. After each layer is cast, the layer must be rinsed in water to remove weakly-attached components before a new layer is input, with hydrophobic and electrostatic effects holding adjacent layers together [72,83,86]. The pace of this process can be greatly increased by adding charged molecules to the solution—which improves polymer deposition rates—and by skipping the rinsing stage by incorporating dimethylformamide within the layering solution [87]. Since the created structure can be finely controlled by modifying its original solution contents, its structure is easily customized for different desirable physical attributes or drug-release rates [72]. Such structures can also be suitable platforms for electrodes and biosensors [4,87].

### 4.5. Embedding

**Figure 6:**
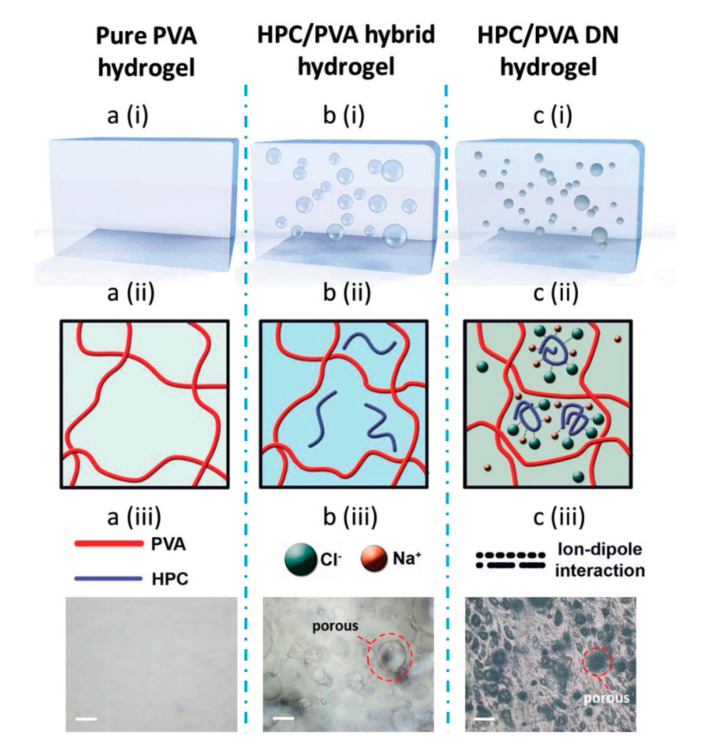
A diagram of three biopolymer-based hydrogels using embedding techniques [88]. (**a**) A pure hydrogel material of polyvinyl alcohol (PVA). (**b**) A hydroxypropyl cellulose (HPC) fiber embedded hydrogel (PVA + HPC). (**c**) A hydrogel embedded with ionically soaked fibrous materials (PVA + HPC + NaCl). Reproduced with permission from Wiley, 2018.

The embedding process creates a composite material for situations where no single material is available to adequately fulfill a given role [88]. Embedding can be performed several different ways, such as dispersing fibrous biopolymers within a hydrogel (Figure 6) [88]. This method merely requires that the fibers be of a small enough length to pass through the nanofibrous matrix prior to introducing the fibers to the hydrogel [89]. Hydrogel-fiber composite materials can alternatively be made by using freeze-thaw cycles to have a solution penetrate a fiber network and exposing the system to crosslinking agents, creating a nanofibrous matrix within a larger fiber bundle with a wide range of mechanical properties controlled by the number of thermal cycles and the initial concentration of monomers [90,91]. Electrical properties may also be controlled by soaking the fibrous components in ionic solutions, prior to embedding [89]. Such composites may be used to create mechanically strong, flexible, electronics or tissue scaffolding [88,89,90]. Other forms of embedding may be performed by mixing a biopolymer solution with quantum dots to create small biosensors [89]—due to the quantum dot’s strong optical properties—or with medications to create drug delivery devices [52,92]. Embedded biopolymers in solution can later be film-cast to provide physical structure to the composite material when necessary [92].

## 5. Applications

**Figure 7:**
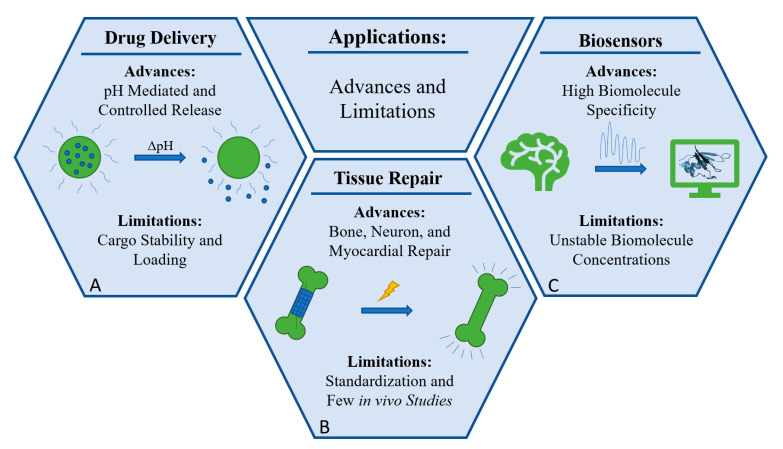
Potential applications of electrically conducting biomaterials are (**A**) pH mediated drug delivery platforms, (**B**) tissue regeneration matrices and (**C**) biosensors. The biosensor example displayed above demonstrates the monitoring of protein concentrations within the brain. The drug delivery diagram showcases pH mediated drug release. The tissue repair diagram shows a biomaterial that, when electrically stimulated, promotes bone regeneration. Major advances and limitations for each technology are also presented as a quick reference.

Advancements in biomaterial research are expanding into the clinic, generating novel therapies and technologies for disease management and treatment. With clinical applications in mind, the ideal biomaterial should be biodegradable and biocompatible. Additional factors for researchers to consider are biomaterial accessibility, conductivity, cytotoxicity, and reproducibility. Four common areas of application include nanomedicine, drug delivery, tissue regeneration and biosensors. Figure 7 presents a general summary for these applications.

### 5.1. Drug Delivery

Advances in drug delivery face many challenges in the coming decades: controlling the release of drugs, delivery biologics in their functional state, and guaranteeing a biocompatible drug delivery vehicle. Biopolymer based material systems are mature drug delivery vehicles, which can be used to deliver various compounds with anti-inflammatory and anti-cancer properties [93,94]. Qu et al. developed a series of injectable conductive hydrogels as smart drug carriers by crosslinking chitosan-graft-polyaniline (CP) copolymer with oxidized dextran (OD). The smart drug delivery system is electrically responsive, has good pH sensitivity and cell compatibility, and inherent antibacterial activity [95]. In a separate study, Kiaee et al. demonstrated advances in a pH dependent drug delivery biomaterial composed of poly (ethylene glycol-diacrylate/Laponite) hydrogel [96]. The material contained chitosan nanoparticles, which are released in low pH environments controlled by DC voltage source. This technology could apply to voltage and pH sensitive areas of the body for targeted drug delivery. Figure 7A displays a schematic representation of pH mediated drug delivery platforms. The large green circle is a voltage sensitive biomaterial and the small blue circles represent drug molecules. When a voltage change occurs, which is a change in pH, the drug carrier releases the cargo. Neumann et al. established a method to enhance the polymer’s ability to function as a drug delivery system in physiologically relevant pH ranges by altering the monomer ratios of the copolymer [97]. Hydrophobic, hydrophilic, and biologically derived drugs showed controlled release with insignificant losses. Additionally, methacrylic acid, ethyl acrylate, and diallyl phthalate cross links behave as insoluble lattices in acidic environments where higher pH environments ionize the polymer, causing electrostatic repulsion and ultimately cargo release [98].

### 5.2. Tissue Regeneration

Tissue engineering and regeneration are important and growing fields aiming to revolutionize medicine [99]. However, translation of laboratory findings to the clinic has been slow. Electrical stimulation of tissue can enhance vascularization of targeted regions and differentiate stem cells into various cells, but targeted and controlled electrical stimulation remains challenging [100,101,102,103,104]. Electrically, conduction biomaterials are an avenue of great promise for overcoming these limitations [105]. For neural tissue engineering, Thunberg et al. electrospun cellulose nanofibers via cellulose acetate and surface modified the materials using a conducting polymer, polypyrole. The biomaterial was nontoxic and supported neural growth with a 10^5^-fold increase in conductivity [106]. In a separate study, Xu et al. produced composite hydrogels comprising carboxymethyl chitosan coated with the conductive polymer poly(3,4-ethylenedioxythiophene). The materials proved biocompatible, fostered cell adhesion and growth, and showed and an electrical conductivity of (4.68 ± 0.28) × 10^−3^ S·cm^−1^ [107]. Cui et al. established an electroactive tissue engineering scaffold made of poly(l-lactic acid)-block-aniline pentamer-block-poly(l-lactic acid) (PLA-AP) with poly(lactic-co-glycolic acid)/hydroxyapatite (PLGA/HA), which is a triblock copolymer with a gene therapy drug pSTAR-hBMP-4 plasmid (phBMP-4) loaded in it. Electrical stimulation releases the drug and improves bone tissue regeneration [108,109]. Figure 7B shows a damaged bone with a blue matrix composed of a voltage sensitive biomaterial. The properties of the matrix upon electrical simulation change, promoting bone repair and regeneration.

### 5.3. Biosensors

Biosensors detect chemicals and other biologics, providing insight to a host of biological processes in vivo such as metabolomic regulation, immune activity, and epigenetic modification [110,111,112]. Looking at electronic skin (E-skin) engineering, Zhang et al. developed a biopolymer by inserting carbon nanotubes into hydroxypropyl cellulose (HPC) and poly (acrylamide-co-acrylic acid) (PACA) composited liquid-crystal hydrogel [108]. The hydrogel E-skins created were highly elastic and conductive, with the ability to make a quantitative response to external stimuli through electrical resistance and visually map the stimulating sites by color changes. Porous synthetic polymer material has gained considerable attention as an optically active biosensor but is limited as far as freshly etched porous polymer matrix is known to generate free radicals and be toxic to living cells. Silk protein and paper (cellulose)-based materials have been shown to overcome these limitations and create renewable and environmentally friendly electronic and photonics devices for biomedical applications [113]. Surface Plasmon Resonance (SPR) has been utilized to investigate the molecular interactions attributed to Alzheimer’s disease because of their ability to detect real time, label free, and highly specific direct detections amongst antibody–antigen, DNA–DNA, DNA–protein, protein–protein, receptor–ligand, and peptide– and protein–membrane interactions [114]. With Alzheimer’s, SPR is used to measure the changes of a propagation constant of localized electromagnetic on a thin layer film attributed to the binding of 14 amino acids from the 17β-HSD10 molecule and hole enzyme 17β-HSD10. Because of the high sensitivity in the changes of refractive index associated with these specific molecules, a highly accurate and non-invasive diagnostic sensor can diagnose Alzheimer’s and prevent further neural cell death. Melanin is another naturally occurring semiconductor that exhibits hydration dependent charge transport signal transductions and naturally degrade within the body. Because of the material electrical properties, the sensor is not only able to recognize charge dependent changes but also induce neurogenesis in vivo. For example, electrospun silk/melanin nanofibrous scaffolds have been developed with superior antioxidant and electroactive properties, which can promote the differentiation and alignment of neuronal cells [115]. These naturally occurring semi-conductors are of interest because they feature high specificity electrical properties but also because of their biocompatibility, making it possible to speed up approval for medical devices. Figure 7C displays brain-implantable biosensors capable of detecting specific proteins, specifically the tau protein associated with Alzheimer’s disease. The protein signals and concentration levels are transmitted to a computer where the results are displayed. The specificity, pertaining to new biomaterials, in targeting disease related changes in local electrical cues will provide society with an advantage in combating disease [116].

## 6. Conclusions

Electrically responsive biomaterials are an important and emerging technology in the fields of biomedical and material sciences. Studies have explored the integral role of electrical conduction in normal and diseased cell biology, and material scientists are focusing a great amount of attention on natural and hybrid protein/polysaccharide materials as sources of biomaterials which can mimic the properties of cells. This review established a summary of those efforts for the latter group, detailing the current materials, theory, methods, and applications of electrically conductive biomaterials. The long-term goal of these studies is to improve human life through novel drug delivery, tissue regeneration, and biosensing technologies. The immediate goal, however, is to establish methods for material creation that are biocompatible and feature adjustable electrical properties. Ideally, these materials will be inexpensive to make with salable production strategies, in addition to being both renewable and biocompatible.

## Data Availability

Not applicable.
